# Hypoxic Brain Injury Mimicking a Spinal Cord Disease: An Unusual Neurological Consequence of Cardiac Arrest

**DOI:** 10.7759/cureus.94265

**Published:** 2025-10-10

**Authors:** Islam Gumaa, Muaiad Mohamed, Maged Kadies

**Affiliations:** 1 Rehabilitation Medicine, Devonshire Centre for Neurorehabilitation, Stockport, GBR; 2 General Surgery, Gadarif Hospital, Gadarif, SDN

**Keywords:** basal ganglia, bilateral lower limb, cardiac arrest, hypoxic-ischemic encephalopathy, watershed ischemic spinal cord stroke

## Abstract

Cardiac arrest remains a major cause of both death and long-term disability, particularly neurological impairment. The well-known complication of cardiac arrest is hypoxic-ischemic encephalopathy (HIE), typically manifesting with altered consciousness and cognitive impairment. We report the rare case of a 51-year-old man who developed bilateral lower limb weakness and neurogenic bladder after an out-of-hospital cardiac arrest due to an inferior ST-segment elevation myocardial infarction (STEMI). Given the clinical presentation, a spinal cord lesion, such as a watershed ischemic spinal cord stroke, was suspected. However, extensive spinal imaging revealed no abnormalities. Instead, brain MRI findings were consistent with HIE, suggesting cerebral hypoxia as the underlying cause of the patient's lower limb dysfunction. This case highlights the importance of considering HIE as a differential diagnosis in post-cardiac arrest patients presenting with lower limb weakness, particularly when spinal imaging is unremarkable. Early recognition is essential for appropriate management and prognosis.

## Introduction

Cardiac arrest (CA) remains a major cause of both death and long-term disability, particularly neurological impairment. Out-of-hospital CA affects approximately 80 individuals per 100,000 annually [1]. Even with advances in resuscitation procedures, only around 10% of these patients survive to hospital discharge, and around 5% fully recover neurologically [1].

The well-known complication of CA is hypoxic-ischemic encephalopathy (HIE), which accounts for 68% of deaths after inpatient CA and 23% of out-of­-hospital CA [2]. HIE commonly presents with altered consciousness, seizures, cognitive and neuropsychiatric symptoms, and autonomic dysfunction. However, it occasionally presents with motor deficits such as quadriparesis or paraparesis, resembling spinal cord injury [3].

Spinal cord ischemia is a rare complication in comparison to cerebral ischemia [4]. The causes of spinal cord injury are varied and include trauma, vascular diseases, aortic surgery, and systemic hypoxic ischemia [5].

Here, we report the case of a man with hypoxic brain encephalopathy who presented with bilateral lower limb weakness following a CA.

## Case presentation

A 51-year-old man with a history of hypertension, anxiety, and smoking presented to the emergency department following an out-of-hospital CA. Upon arrival at the hospital, cardiopulmonary resuscitation (CPR) was initiated, and the patient received two defibrillator shocks and multiple doses of adrenaline. The total downtime was approximately 30 minutes before the return of spontaneous circulation. ECG showed an inferior ST-segment elevation myocardial infarction (STEMI) complicated by ventricular tachycardia as a cause of the CA. Therefore, he had an angiogram that showed an intense right coronary artery vasospasm of unclear etiology. This resolved with glyceryl trinitrate (GTN), but then he developed a reperfusion rhythm requiring two cardioversion shocks and was intubated and transferred to cardiothoracic critical care for further care. Once his consciousness improved, he was found to have reduced power in both lower limbs and increased tone all over the lower limbs with increased reflexes; however, all sensation modalities are intact. Neurological examination is shown in Tables 1-2.

**Table 1:** Lower limb motor examination

Range of movement and strength	Left lower limb	Right lower limb
Hip flexion	0/5	1/5
Hip extension	0/5	1/5
Hip abduction	2/5	2/5
Hip adduction	1/5	1/5
Knee flexion	2/5	0/5
Knee extension	2/5	0/5
Ankle dorsiflexion	1/5	1/5
Ankle plantar flexion	1/5	1/5

**Table 2:** Upper limb motor examination

Range of movement and strength	Left upper limb	Right upper limb
Shoulder flexion	4+/5	5/5
Shoulder extension	5/5	5/5
Shoulder abduction	5/5	5/5
Shoulder adduction	5/5	5/5
Shoulder medial rotation	5/5	5/5
Shoulder lateral rotation	5/5	5/5
Elbow flexion	5/5	5/5
Elbow extension	5/5	5/5
Forearm pronator	5/5	5/5
Forearm supinator	5/5	5/5
Wrist flexion	5/5	5/5
Wrist extension	5/5	5/5
Wrist ulnar deviation	5/5	5/5
Wrist radial deviation	5/5	5/5

**Figure 1:**
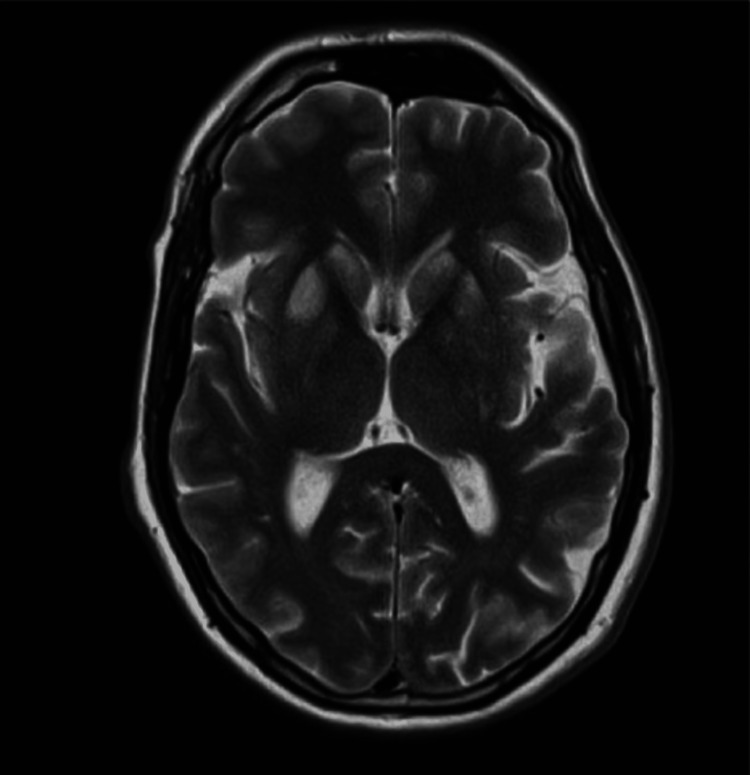
T2-weighted MRI scan showing bilateral symmetrical T2/FLAIR signal abnormality within the bilateral head of the caudate, globus pallidus, and putamen, evidence of restricted diffusion from the region of the globus pallidus on DWI FLAIR: fluid-attenuated inversion recovery; DWI: diffusion-weighted imaging

**Figure 2:**
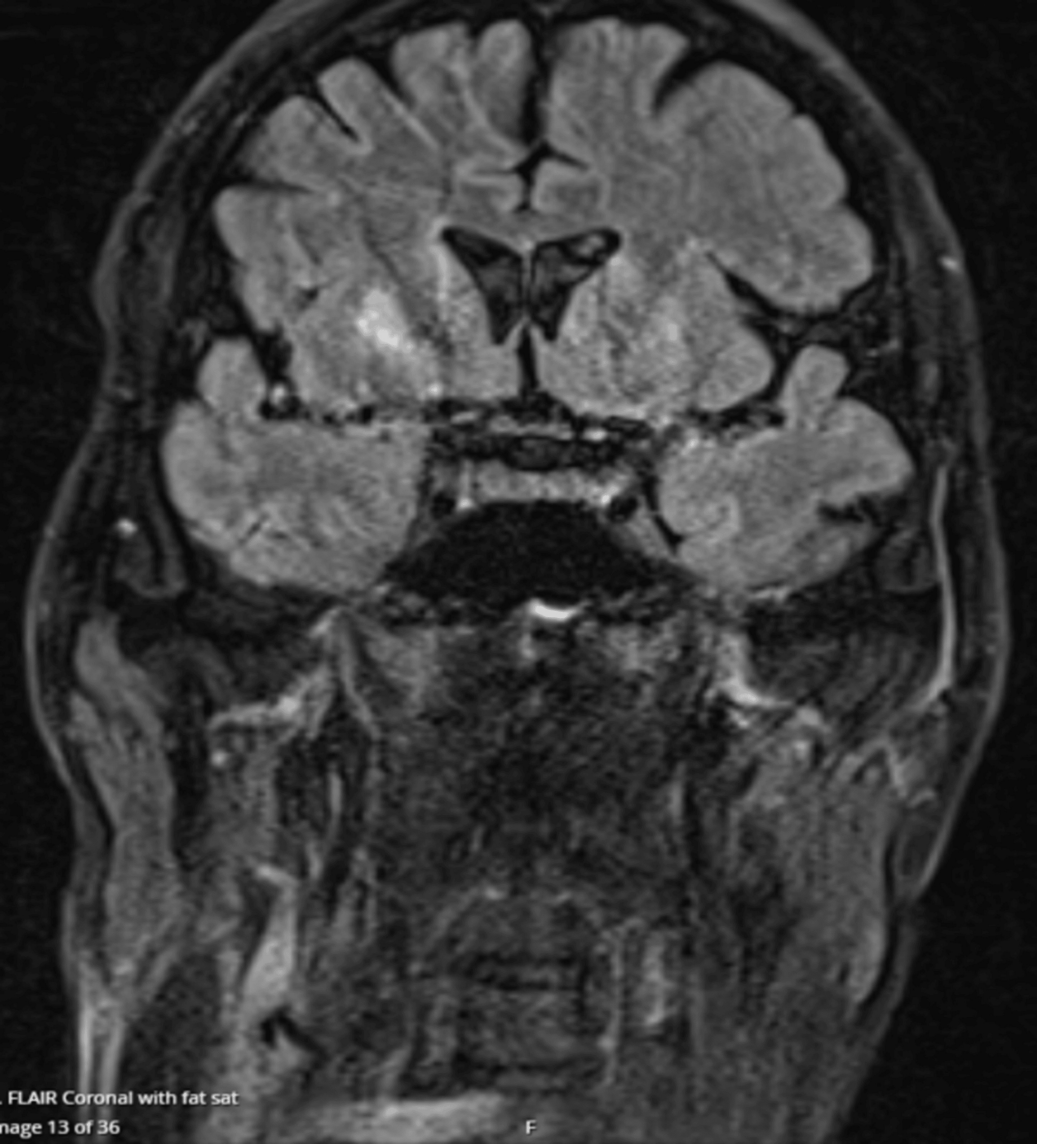
T2-weighted MRI scan

Moreover, the patient lacked bladder sensation, was incontinent, and required catheterization. The Glasgow Coma Scale (GCS) score was 15; however, there were issues with cognition, in particular, slow information processing, some short-term memory difficulties, and an inability to recall information. The upper limb examination was completely normal. Eventually, an MRI was performed at the recommendation of the neurologist, who felt that clinically this presentation was likely to be a watershed ischemic spinal cord stroke secondary to the CA. He had two MRI spine examinations, both of which were normal and did not show any evidence of spinal cord infarct. Later, as his neurological deficit persisted, an MRI of the head was requested and showed bilateral symmetrical T2/fluid-attenuated inversion recovery (FLAIR) signal abnormality within the bilateral head of the caudate, globus pallidus, and putamen, evidence of restricted diffusion from the region of the globus pallidus on diffusion-weighted imaging (DWI). Appearances were strongly suspicious of HIE given the recent history of out-of-hospital CA with prolonged downtime and no acute brain ischemia or hemorrhage, as shown in Figures 1-2. Once clinically stable, the patient was transferred to an inpatient neurorehabilitation unit for further therapy. 

**Figure 3:**
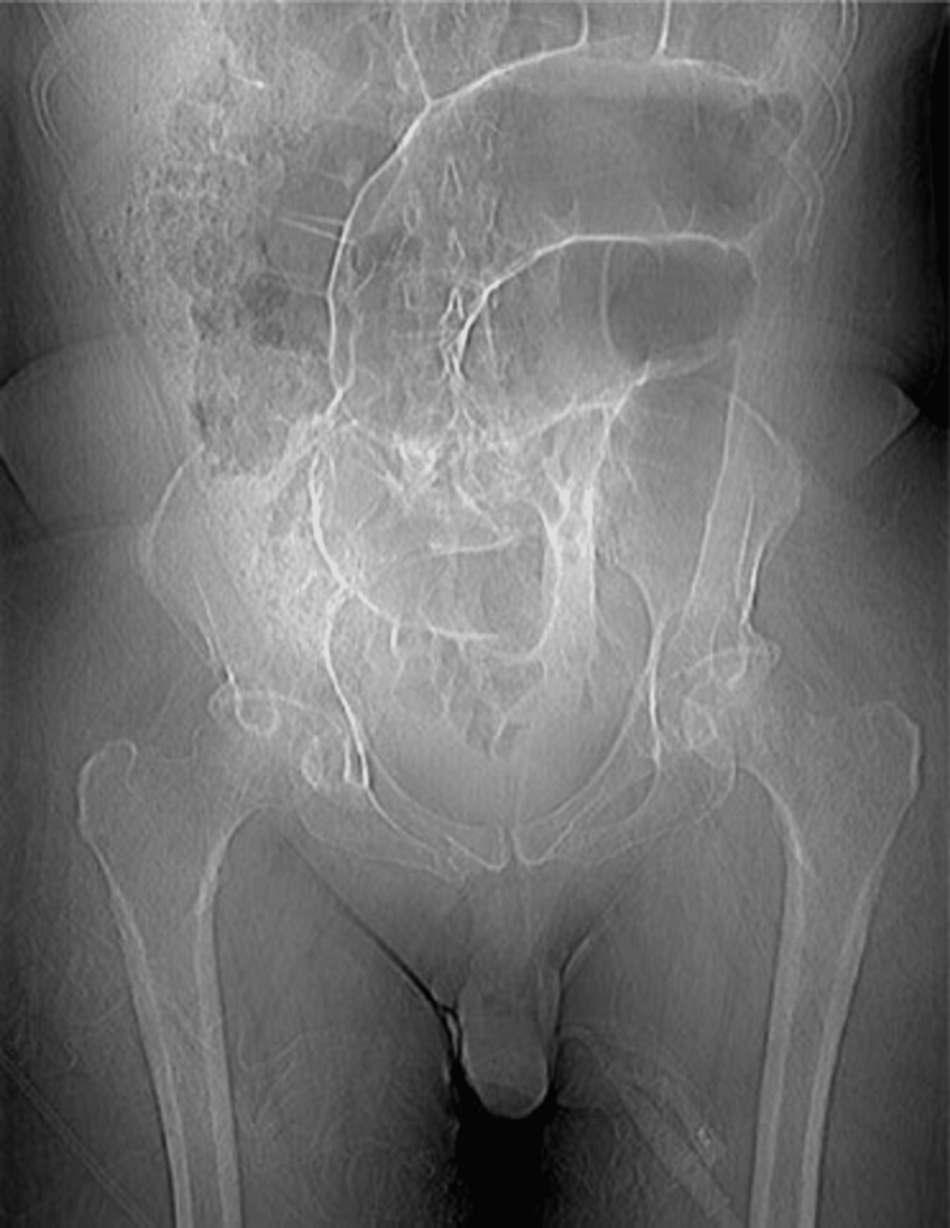
CT of the abdomen with contrast showing bowel distension and not revealing any specific pathology

**Figure 4:**
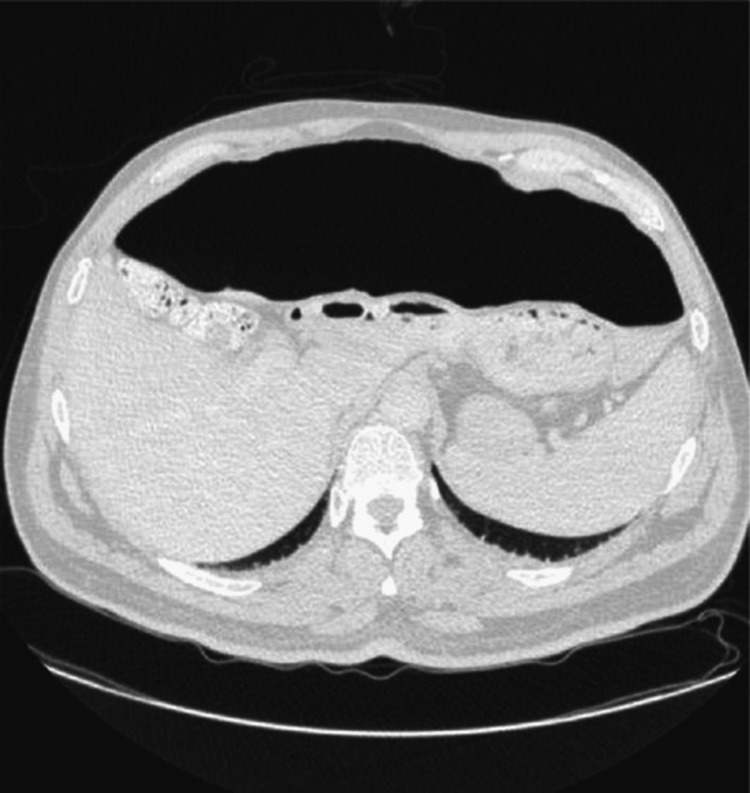
CT of the abdomen with contrast showing bowel distension and no other pathology

**Figure 5:**
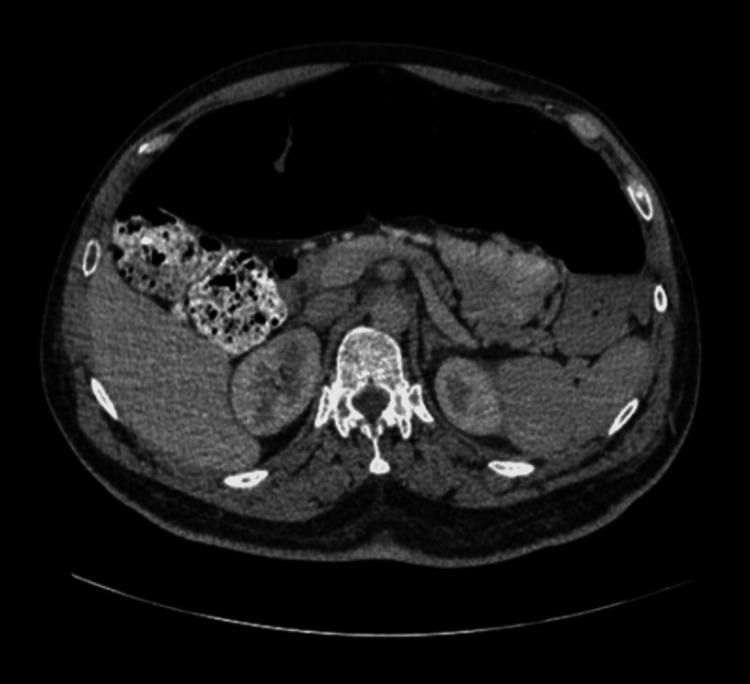
CT of the abdomen with contrast showing bowel dysmotility and distension

During admission, physiotherapy colleagues noted that this patient had more tone in the lower limbs, given the degree of weakness when compared to patients with purely spinal pathology. Moreover, it was noted that a patient's leg tone varied greatly in response to anxiety and stress. He remained an inpatient in a neurorehabilitation unit for seven months, making slow and very limited progress in his mobility. At the time of the case conference, six months into admission, the patient was able to transfer independently but remained reliant on a wheelchair for mobility. During admission, the patient developed a distended abdomen and diarrhea. Multiple investigations, including a CT of the abdomen, did not reveal any specific pathology other than bowel distension and dysmotility as shown in Figures 3-5. It was felt that this was a neurogenic bowel secondary to HIE. 

## Discussion

In this case report, we described a patient who presented predominantly with lower limb weakness following a prolonged CA. The clinical presentation of the patient initially suggested spinal cord infarction, but subsequent neuroimaging revealed basal ganglia changes consistent with cerebral HIE.

Neurological complications are common among patients surviving CA. However, the pattern of neurological deficits can differ significantly according to the duration and severity of hypoxia and the involvement of specific brain regions. The cerebral cortex, hippocampus, and basal ganglia are highly sensitive to hypoxia [6]. A well-documented consequence of prolonged CA is HIE, which leads to diffuse neurological deficits. A study done by Endisch et al. demonstrated that severe HIE is present in 61% of post­-CA patients upon brain autopsy [7]. However, our case is unique in that the patient predominantly exhibited lower limb weakness and spasticity, similar to spinal cord injury rather than global encephalopathy. Moreover, this case manifested bladder dysfunction and cognitive impairment, all of which suggest considerable brain injury.

The initial suspicion in this case was spinal cord infarction because of the marked lower limb weakness and preserved upper limb function. The majority of spinal cord infarcts happen in watershed zones, particularly in the thoracic cord, where the blood supply is relatively weak [8]. However, the absence of MRI changes of the spinal cord after repeated imaging led to a reconsideration of the diagnosis. The subsequent brain MRI, revealing basal ganglia abnormalities, confirmed the cerebral origin of the patient's symptoms. The selective bilateral involvement of the basal ganglia in our case, especially the caudate, globus pallidus, and putamen, on MRI goes with what was reported by Ghei et al. [9]. These deep grey matter structures are more vulnerable to hypoxic-ischemic injury because of their increased metabolic demand, dense glutamatergic input, and limited collateral perfusion compared with cortical regions.

Another possible differential diagnosis includes toxic or metabolic causes of basal ganglia dysfunction, which can resemble hypoxic injury. For example, as reported by Weaver, carbon monoxide poisoning selectively damages the globus pallidus and can manifest as confusion, memory deficits, and motor impairment resembling HIE [10].

The case's predominant involvement of the lower limb aligns with what has been described as selective vulnerability patterns in HIE. The basal ganglia are particularly liable to hypoxic injury because of their high metabolic demands and watershed vascular supply [11]. The basal ganglia are frequently involved in motor control dysfunction after hypoxic brain injury, resulting in movement disorders such as hypertonia and rigidity as reported by Scheibe et al. [12]. Stress or anxiety increases the patient's lower limb tone, which aligns with the literature, where emotional situations often worsen hyperkinetic or hypertonic symptoms in individuals with basal ganglia injury [12]. These structures are essential for motor control, and their injury can manifest as lower limb predominant symptoms due to somatotopic organization in these areas.

Despite the prolonged rehabilitation course, the patient's mobility has seen minimal improvement after seven months. This is in keeping with the literature suggesting that patients with basal ganglia involvement following HIE have poor outcomes. Heinz and Rollnik reported that about three-quarters of HIE patients had a poor outcome, defined as a Barthel Index (BI) score below 50. Notably, all patients exhibiting bilateral basal ganglia hypodensities had poor outcomes [13].

An important limitation is that although the patient's MRI findings showed bilateral basal ganglia hyperintensities characteristic of HIE, the possibility of concurrent spinal cord ischemia cannot be completely excluded. In our case, the acute onset of paraplegia and urinary dysfunction following resuscitation is compatible with anterior spinal artery involvement; however, both spinal MRI scans reported no abnormalities. It is essential to recognize that early or mild spinal cord infarction may not always be visualized on MRI, particularly within the first 24-48 hours, as diffusion and T2-weighted signal changes can lag behind clinical manifestations [14]. Therefore, the absence of radiological abnormalities does not definitively exclude spinal ischemia. This limitation emphasizes the diagnostic challenge in the differentiation between spinal cord infarction and hypoxic-ischemic brain injury in post-CA patients, highlighting the need for serial imaging and correlation with the clinical picture.

## Conclusions

This case highlights the significance of comprehensive neuroimaging in patients with focal neurological deficits post-CA. While the initial clinical impression may be suspicious of spinal pathology, cerebral causes, including selective basal ganglia injury, should be considered. Also, it emphasizes the importance of neurorehabilitation in managing persistent neurological deficits post-HIE, even in cases with unusual presentations. Moreover, it asserts the need for clinicians to identify this pattern as an atypical presentation of post-anoxic brain insult in the situation of prolonged CA.
